# Comparison of Anatomical Conformity between TomoFix Anatomical Plate and TomoFix Conventional Plate in Open-Wedge High Tibial Osteotomy

**DOI:** 10.3390/medicina58081045

**Published:** 2022-08-03

**Authors:** Sung-Sahn Lee, Jaesung Park, Dae-Hee Lee

**Affiliations:** 1 Department of Orthopedic Surgery, Ilsan Paik Hospital, School of Medicine, Inje University, 170 Juwha Street, Goyangsi 10380, Korea; sungsahnlee@gmail.com; 2 Department of Orthopedic Surgery, Samsung Medical Center, School of Medicine, Sungkyunkwan University, 81 Irwon Street, Seoul 06351, Korea; jspark3168@gmail.com

**Keywords:** high tibial osteotomy, TomoFix, plate position, anatomical conformity

## Abstract

*Background and Objectives*: The TomoFix anatomical plate was developed to improve plate position, proximal screw direction, and post-correction tibial contouring. The purpose of this study was to compare postoperative configurations between the TomoFix anatomical plate and the TomoFix conventional plate. It was hypothesized that the new modified plate provides a better fixative coaptation than the conventional plate. *Materials and Methods*: A total of 116 cases (112 patients) were enrolled in this study from March 2015 to February 2021. Among them, 63 patients underwent surgery using the TomoFix conventional plate, and 53 underwent surgery using the TomoFix anatomical plate. The radiographic outcomes, including the hip–knee–ankle (HKA) angle, medial proximal tibial angle (MPTA), tibial slope, plate angle, proximal screw angles, and plate-to-cortex distance at #1 hole (just below the osteotomy site) were compared between the two groups. *Results*: Patients with the TomoFix anatomical plate showed similar results in terms of the pre- and postoperative HKA angle, MPTA, and tibial slope. The TomoFix anatomical group showed a significantly greater plate angle (39.2° ± 8.1° vs. 31.7° ± 7.0°, *p* < 0.001) and less screw angles, indicating that the TomoFix anatomical plates allowed a more posterior plate position than the conventional plate. The plate-to-cortex distance was significantly less in the TomoFix anatomical group than in the TomoFix conventional group (*p* < 0.001). *Conclusion*: The TomoFix anatomical plate showed a more posteromedial plating position, better proximal screw direction to the lateral hinge, and improved post-correction tibial contour compared to the TomoFix conventional plate.

## 1. Introduction

High tibial osteotomy is a well-established surgical procedure for the management of medial unicompartmental arthritis of the knee joint [1,2,3,4]. A lateral closed-wedge high tibial osteotomy has potential disadvantages, including difficulty in intraoperatively controlling the amount of correction and concerns of neurologic deficits [5,6]. Due to these disadvantages, open-wedge high tibial osteotomy (OWHTO) is becoming popular [7].

The mechanical stability of the osteotomy site is a major issue following OWHTO. Correction loss and non-union of the osteotomy site have been observed after surgery [8,9,10]. However, with the introduction of the TomoFix plate (Synthes GmbH, Oberdorf, Switzerland), which is characterized by a T-shaped, long, rigid-angle stable implant, the incidence of non-union following OWHTO has declined significantly [8,9].

**Figure 1:**
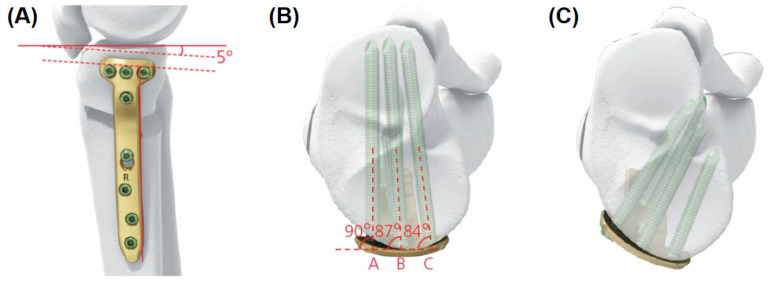
(**A**) Schematic diagram of the new plate lateral view. While the previous plate was a 90° T-shaped plate with no left and right distinction, the TomoFix anatomical plate has a 5° posterior slope, allowing the plate to be positioned relatively backward. As a result, the new plate system (**B**) enables parallel screw fixation than the previous plate (**C**).

Plate position and proximal screw angles are important for fixation stability in OWHTO [11]. Posteromedial plating provides better fixation stability than anteromedial plating [11,12]. The proximal screw in the direction of the lateral hinge rather than the posterior tibial cortex is associated with increased stability and less neurovascular injury [12]. Anatomical contouring to the post-correction medial tibial geometry is also important for decreasing the distance between the plate and cortex [13,14]. The TomoFix anatomical plate was developed to improve plate position, proximal screw direction, and post-correction tibial contouring (Figure 1).

Therefore, the purpose of the current study is to compare postoperative configurations between the new modified plate and the conventional plate (the TomoFix Anatomical plate versus the TomoFix conventional plate). It was hypothesized that the new modified plate would provide a better anatomical fitness than the conventional plate.

## 2. Materials and Methods

This study enrolled patients with primary medial osteoarthritis who underwent primary OWHTO between March 2015 and February 2021. The included subjects were patients aged <65 years who had osteoarthritis with persistent pain despite conservative management for more than three months. Patients were excluded if they had severe osteoarthritis of the patellofemoral joint or lateral compartment, ligament laxity, rheumatoid arthritis, or flexion contracture > 15°, and required an angle of correction > 20°. A total of 116 cases (112 patients) were enrolled in the current study. Among them, 63 patients underwent surgery using the conventional TomoFix plate and 53 using the TomoFix Anatomical plate. For patients who underwent surgery between March 2015 and July 2018, OWHTO was performed using the TomoFix conventional plate, after which surgery was performed using the TomoFix anatomical plate. Both plates were made of titanium. The patient demographics and preoperative data are summarized in Table 1. This study was approved by the Ethics Committee of our institution (SMC2021-05-051 at 26 May 2021), and written informed consent was obtained from all patients.

### 2.1. Surgical Technique

All surgeries were performed by the senior author. The correction angle was measured using Miniaci’s method [15,16]. The alignment of the lower limb was adjusted by passing the point located at 62.5% of the tibial plateau width when measured from the edge of the proximal tibial medial plateau [17]. Biplanar osteotomy was performed in all knees. After skin incision, a guide wire was inserted with visual assistance by an image intensifier. The oblique part of the osteotomy was performed with a saw for a distance of up to 1 cm from the lateral cortex. The osteotomy of the vertical part was performed at the posterior aspect of the tibial tubercle, thus not violating the bony portion to which the patellar tendon was attached, leaving most of the tendon attached to the distal tibial fragment. The anterior gap of the osteotomy sited behind the tibial tuberosity was intended to be approximately 1/2 to 2/3 of the posterior opening gap at the posteromedial corner of the proximal tibia to maintain tibial slope. Target alignment was achieved under intraoperative fluoroscopy, and the osteotomy was stabilized using the TomoFix conventional plate or the TomoFix anatomical plate. A cortical screw was used on the most proximal hole of the distal holes (the so-called #1 hole), if the bone-to-plate distance was more than 5 mm, to avoid fixative failure [18]. An allogenic bone graft (Junyoung Medical, Seoul, South Korea) was inserted into the osteotomy gap to minimize postoperative loss of correction.

The patients were started on isometric quadriceps, active ankle, and straight leg-raising exercises on the day after surgery. Knee motion exercises were initiated on the second postoperative day. Patients were restricted toe toe-touch weight bearing for the first 2 postoperative weeks, followed by partial weight bearing for next 4–6 weeks. Full weight-bearing was permitted by 6–8 weeks.

### 2.2. Radiographic Evaluation

Plain radiographs were obtained before surgery and three months postoperatively. The radiographs included whole leg standing radiographs (patella facing forward and full knee extension) and lateral views with 30° flexion. The change in limb alignment from before to after surgery was determined by measuring the hip–knee–ankle (HKA) angle on full-length standing radiographs, using a picture archiving and communication system (Centricity; General Electric, Chicago, IL, USA) [19,20]. The HKA angle was measured by the angle subtended by a line drawn from the center of the femoral head to the center of the knee and a line drawn from the center of the knee to the center of the talus. The medial proximal tibial angle (MPTA) was measured as the angle between the proximal anatomical axis of the tibia and the tangent along the articular surface of the tibial plateau on full-length standing radiographs [21]. The angle of the tibial slope was defined using the proximal tibial anatomical axis method [19]. The MPTA and tibial slope were also compared before and after surgery and between both plate systems.

**Figure 2:**
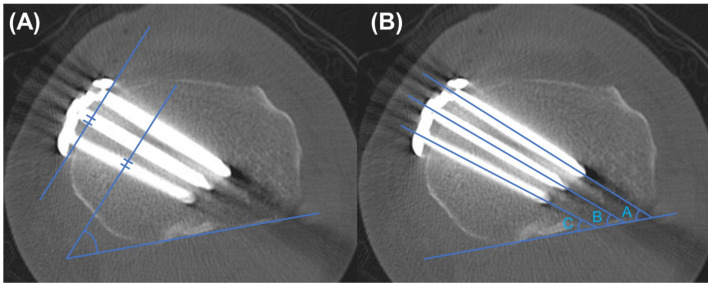
Measurement of the plate angle and screw angles on the CT axial scan. (**A**) The plate angle was defined as the angle between the anteroposterior line connecting the proximal part of the plate and the posterior tibial condylar line. (**B**) The A, B, and C screw angle was defined as the angle between the line drawn along the screw and the posterior tibial condylar line. (A, B, and C screws from anterior to posterior, respectively).

**Figure 3:**
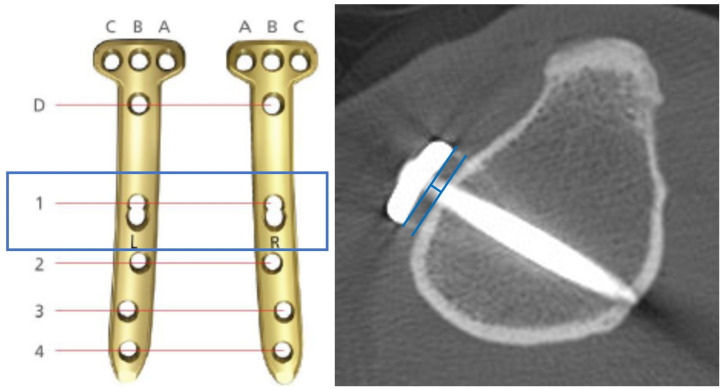
Measurement of the plate-to-cortex distance at #1 hole. It was defined as the distance between the inner surface of the plate and the outer surface of the tibial cortex at #1 hole.

Computed tomography (CT) scans (MDCT; Brilliance 64, Phillips, Cleveland, OH) were performed on the fifth postoperative day. The proximal plate position was evaluated by the proximal plate angle on the axial CT images [14]. The proximal plate angle was defined as the angle between the posterior cortex line and the line connecting the front and back of the plate proximal area (Figure 2A). The most proximal screw (A screw, B screw, and C screw, each from front to back, respectively, Figure 2B) angles were also measured on axial CT images. Each screw angle was defined as the angle between the posterior cortex and the line drawn along the screw. The plate-to-cortex (just below the osteotomy site) distance at #1 hole was measured, and the usage of cortical screw on #1 hole was counted to evaluate the post-correction tibial contouring of the plate (Figure 3).

All measurements were performed by two orthopedic surgeons with significant experience in OWHTO, but who were not associated with the subject of this study to assess interobserver reliability, and were repeated six weeks later to assess intra-observer reliability.

### 2.3. Statistical Analysis

The *Shapiro–Wilk* test was used to investigate the normality of distribution. The *Student’s t*-test was used for continuous variables, whereas the *chi-squared* test was used for categorical variables to compare the demographic data and radiographic data. Statistical significance was set at *p* < 0.05. All data were analyzed using SPSS software (version 22, IBM Corp, Armonk, NY, USA). To have a 90% probability of detecting a 1 mm difference in the plate-to-cortex distance, we needed to enroll 72 patients, assuming an overall standard deviation in 2 mm and a 2-tailed alpha-level of 5%.

## 3. Results

All inter- and intra-observer intraclass correlation coefficients showed good agreement for all radiographic measurement reliabilities (>0.80).

Compared to the TomoFix conventional group, the TomoFix anatomical group showed similar results in terms of sex distribution, mean age, body mass index, and preoperative and postoperative simple radiographic measurements (Table 2).

With respect to CT scan measurements, the TomoFix anatomical group showed a greater plate angle than the TomoFix conventional group (39.2° ± 8.1° vs. 31.7° ± 7.0°, *p* < 0.001, Table 3). This result indicates that the TomoFix anatomical plates allowed a more posterior plate position than the conventional plate. Moreover, all screw angles were significantly lower in the TomoFix anatomical group. These results were also caused by positioning the plate more posteriorly than in the TomoFix conventional group.

In the TomoFix conventional group, a cortical screw on #1 hole (the most proximal hole of distal 4 holes) was used in 16 cases (25.4%). However, cortical screws were used in eight cases (15.1%) in the TomoFix anatomical group. The anatomical plate group used fewer cortical screws on #1 hole, but the difference was not statistically significant (*p* = 0.18). The plate-to-cortex distance was significantly shorter in the TomoFix anatomical group than in the TomoFix conventional group regardless of the use of a cortical screw on the #1 hole. (*p* < 0.001).

## 4. Discussion

The principal finding of the current study was that the TomoFix anatomical plate represented a more posteromedial plating position, proximal screw toward the lateral hinge rather than posterior cortex, and improved post-correction tibial contour compared to the TomoFix conventional plate.

Numerous studies have demonstrated that posteromedial plating provides better fixative stability than anteromedial plating in OWHTO [11,12,22]. Takeuchi et al. [11]. compared the stress on the TomoFix plate between the anteromedial and medial plating positions. They reported that the medial plate position provided significantly less stress on the plate than the anteromedial plate position. Moreover, previous studies reported that the anterior plate position was associated with an increased posterior tibial slope, which might lead to anterior cruciate ligament damage after OWHTO [23,24]. The TomoFix conventional plate was a 90° T-shaped plate with no left and right distinction, while the TomoFix anatomical plate had a 5° posterior slope, allowing the plate to be positioned relatively backward. In the current study, the patients using the TomoFix anatomical plate showed a significantly posterior position compared to those using the conventional plate among single-surgeon cases. These results are consistent with the intent of the TomoFix anatomical plate design.

It is well known that an increased plate–bone distance might reduce the fixative stability when using a locking compression plate and screws. Ahmad et al. [18]. demonstrated that, 5 mm from the bone, the locking plate exhibited increased plastic deformation during cyclical compression and required lower loads to induce mechanical failure when compared to a lesser distance between the bone and plate. Therefore, post-correction anatomical contouring is important for reducing the plate–bone distance in OWHTO. The plate-fitting technique using cortical lag screws was widely performed [25]. However, it might induce a change in the opening gap or lateral hinge fracture [26]. In the current study, in the patients with the TomoFix anatomical plate, fewer cortical screws were used on #1 hole (15.1% vs. 25.4%, *p* = 0.128). Moreover, the plate-to-cortex distances were significantly shorter in the TomoFix anatomical group regardless of the use of a cortical screw. These results demonstrated that the TomoFix anatomical plate showed better anatomical contouring to the post-correction tibia.

OWHTO is a promising surgery for medial compartmental knee joint osteoarthritis. However, adverse events were reported following OWHTO in 37% to 55% of cases [25,27,28]. Sidhu et al. [29] demonstrated that a low rate of serious complications (6.5%) requiring unplanned additional surgery was noted after OWHTO using a TomoFix locking plate. However, 52% of the knees required elective hardware removal due to soft tissue irritation. The TomoFix anatomical plate is expected to have a lower hardware removal rate as it has a more posterior position and is more suitable for the post-correction anatomical structure of the tibia.

This study has some limitations. First, although the TomoFix anatomical plate provided a better configuration than the previous version plate system, the superiority of the actual mechanical stability was not proven. An investigation including a mechanical test is needed to identify the association between fixative configuration and mechanical stability in the future. Second, the current study did not include clinical outcomes, including patient-reported outcome measurements, union rate, union period, and correction loss rate. Therefore, this study was not able to identify the effects of plate design changes on clinical outcomes. Third, the two groups had OWHTO performed at different times (TomoFix conventional plate was used between March 2015 and July 2018, TomoFix anatomical plate was used between August 2018 to February 2021), which may have affected the outcome. However, this study was based on data from a single surgeon, which would have had little effect on the results.

## 5. Conclusions

The TomoFix anatomical plate showed a more posteromedial plating position, proximal screw direction toward the lateral hinge rather than posterior cortex, and improved post-correction tibial contour compared to the TomoFix conventional plate.

## Figures and Tables

**Table 1:** Demographic and preoperative data.

Number of Cases, *n*	116
Sex, M:F	35:81
Age, year	56.9 ± 7.0 (43–77)
BMI, kg/m^2^	27.5 ± 3.4 (20.4–38.0)
Direction, R:L	50:66
Preoperative HKA angle, °	8.6 ± 3.0 (5–20.1)
Preoperative MPTA, °	84.6 ± 2.0 (79.5–89.3)
Preoperative tibial slope, °	10.1 ± 3.7 (1.7–20.7)

BMI, body mass index; HKA, hip-knee-ankle; MPTA, medial proximal tibial angle.

**Table 2:** Comparison of the demographic and simple radiographic data between the TomoFix conventional plate and the TomoFix anatomical plate groups.

	TomoFix Conventional	TomoFix Anatomical	*p*-Value
Number of cases, *n*	63	53	
Sex, M:F	17:46	18:35	0.415
Age, year	56.0 ± 7.4	57.8 ± 6.5	0.171
BMI, kg/m^2^	27.4 ± 3.3	27.7 ± 3.5	0.681
Direction, R:L	28:35	22:31	0.851
Preoperative HKA angle, °	8.3 ± 2.7	9.0 ± 3.3	0.156
Preoperative MPTA, °	84.8 ± 2.2	84.3 ± 1.9	0.26
Preoperative tibial slope, °	10.4 ± 3.7	9.7 ± 3.6	0.308
Opening width, mm	10.6 ± 2.6	11.2 ± 1.7	0.169
Postoperative HKA angle, °	-3.1 ± 2.5	-2.5 ± 2.3	0.135
Postoperative MPTA, °	92.8 ± 2.5	92.5 ± 2.5	0.469
Postoperative tibial slope, °	11.8 ± 4.2	11.4 ± 3.8	0.549

BMI, body mass index; HKA, hip–knee–ankle; MPTA, medial proximal tibial angle.

**Table 3:** Comparison of the fixative device position on CT scan between the two groups.

	TomoFix Conventional	TomoFix Anatomical	*p*-Value
Plate angle, °	31.7 ± 7.0	39.2 ± 8.1	0.001
A screw angle, °	61.4 ± 6.6	51.8 ± 7.4	<0.001
B screw angle, °	57.8 ± 7.3	49.6 ± 7.7	<0.001
C screw angle, °	54.2 ± 7.4	46.6 ± 7.4	<0.001
Using cortical screw on #1 hole	16 cases (25.4%)	8 cases (15.1%)	0.128
Plate-to-cortex distance (all cases), mm	3.2 ± 1.7	1.7 ± 1.3	<0.001
Plate-to-cortex distance (only using locking screw), mm	3.9 ± 1.1	1.9 ± 1.2	<0.001

## Data Availability

Not applicable.
